# Dietary determinants of overnutrition among hypertensive patients in southwest Ethiopia: an ordinal regression model

**DOI:** 10.1038/s41598-024-57496-y

**Published:** 2024-04-02

**Authors:** Melaku Gebre, Girma Alemayehu Beyene, Ebrahim Muktar, Amare Zewdie, Agize Asfaw, Abebaw Wasie Kasahun, Abdurezak Kemal, Abdu Oumer

**Affiliations:** 1 Department of Public Health, College of Medicine and Health Sciences, Wolkite University, Gubre, Ethiopia; 2 Department of Health, Behavior and Society, Faculty of Public Health, Institute of Health, Jimma University, Jimma, Ethiopia; 3 School of Public Health, College of Medicine and Health Sciences, Haramaya University, Harar, Ethiopia

**Keywords:** Dietary pattern, Food frequency questionnaire, Hypertension, Obesity, Overnutrition, Epidemiology, Translational research, Nutrition, Public health

## Abstract

Overnutrition is a recognized risk factor for hypertension, but evidence is lacking among hypertensive patients for tailored dietary interventions. This study assessed dietary factors in 331 hypertensive patients in southwest Ethiopia. The data was collected through a questionnaire and analyzed using factor analysis. Body mass index (BMI) was calculated, and a BMI above 25 kg m^−2^ was considered overnutrition. An ordinal logistic regression model was used to model the data and control confounders. Adjusted odds ratio and *p*-values were reported. Among the 331 respondents, consumption of cereals and grains (57.0%); roots and tubers (58.5); and legumes (50.0%), while 28.6% drink alcohol, was common. About 29.0% (24.1–34.2) had overnutrition (22%, 17.6–26.6%, overweight and 7.0%, 4.5–10.3%, obesity). While the predicted odds of overnutrition were higher among males (AOR = 2.85; 1.35–6.02), married (AOR = 1.47; 0.69–3.12), illiterates (AOR = 2.09; 1.18–3.72), advanced age (AOR = 1.65; 0.61–4.61), government employees (AOR = 6.83; 1.19–39.2), and urban dwellers (AOR = 4.06; 1.76–9.36), infrequent vegetable consumption (AOR = 1.47; 0.72–2.96) and lower and higher terciles of cereals and animal-source food consumption (AOR = 1.56; 0.72–3.34). Overnutrition among hypertensive patients was significantly high and associated with unhealthy dietary consumption, educational status, residence, and occupation, emphasizing the need for targeted dietary counseling.

## Introduction

Hypertension is a major public health problem and the leading cause of cardiovascular diseases, contributing to 9.4 million deaths and 1.39 billion cases annually^1,2^. These could be associated with the rise in obesity^3^ and other treatment-related factors^4^. Evidence has also shown that the risk of hypertension rises by 3–7 times among those who are overnourished^5,6^, and more than 65% of hypertension is attributable to having excess weight^7,8^. On the contrary, weight loss of 5–10 kg could substantially lower the risk of hypertension by 25%^3^, emphasizing the need for optimal weight for having normal blood pressure.

Overnutrition encompassing overweight and obesity among adults is one of the established risk factors for many non-communicable diseases. It is diagnosed with a higher body mass index between 25 to 29.9 kg m^−2^ (overweight) and above 30 kg m^−2^ (obese)^9^. Overnutrition is becoming alarming global public health challenge^2,10^. There is a rising prevalence of overweight, obesity, and hypertension in both developing and developed countries^2,11^, mainly due to changing dietary habits and sedentary lifestyles^8,12^. Overnutrition is considered as the major non-infectious epidemic, and the second leading cause of preventable death globally^13^, affecting 2 billion adults^14^, and causing nearly 2.8 million annual deaths. The obesity epidemic is spreading to low- and middle-income countries (LMICs) as a result of new dietary habits and sedentary ways of life, fueling chronic diseases and premature mortality^5,7^. Despite the fact that overweight and obesity are health problems in high-income countries, LMICs, in particular urban settings in sub-Saharan African countries, face the greatest challenge^15^ and are under steady increment^16^. Moreover, the prevalence of overweight and obesity was different from region to region, ranging from 19.5 to 24.5%^17^, where 18.4% of women and 7.8% of men are victims of obesity in Ethiopia^18^.

The likelihood and severity of hypertension are closely related to their BMI^19^. It is clear that obesity is a risk factor for hypertension^18^ and can determine treatment success among hypertensive patients. A large-scale study conducted in Bandung indicated that overnutrition among hypertensive patients is highly predictive of cardiovascular complications and deaths by 1.41–5.79 times^20^. Although overnutrition and body fat accumulation is established risk factor for hypertension, there is lack of clear epidemiological evidence linking diet assessed in more comprehensive manner in the study context, which would be very helpful for tailored behavioral interventions. Hence, the usual methods of dietary exposure assessment like individual nutrient and food intake assessment approaches, are not comprehensive and holistic and ultimately predict disease outcomes in a confounded manner. Hence, we tried to apply a more robust statistical modeling to derive the likely dietary consumption patterns using “dietary pattern analysis”^21^. This has been shown to be more informative and predictive in previous studies for predicting the risk of overnutrition and central obesity among the general population^22,23^.

Evidence also depicts that adherence to healthy dietary patterns rich in fruits, vegetables, cereals and legumes, is an important component in the prevention of hypertension and could determine the risk of overnutrition. The current study could help to further refine the existing dietary recommendations for chronic disease care and attain reasonable treatment success. Moreover, previous studies treated the outcome as binomial while the data is ordinal in nature, where we have used a more robust and appropriate method, ordinal regression^24^. Hence, the current study was to quantify the magnitude of overnutrition and identify relevant dietary determinants of overnutrition among hypertensive patients in southwest Ethiopia.

## Results

### Socio-demographic and socio-economic characteristics

From the total of 338 individuals with hypertension invited, 331 participated in our study, with a response rate of 98.0%. The majority of the participants (54.3%) were males, married (80%), Gurage (79.0%), had no education at all (54.0%), and 58% lived in a rural setting. Regarding the religious affiliation of study participants, 44.1% were Muslims, followed by Orthodox Christians (36.2%). Most of the participants, 31.0%, were farmers, and 75.0% were categorized as low-income. The mean family size of the participants was 5.4 (± 2.1) (Table 1).

**Table 1:** Socio-demographic, economic clinical characteristics of Hypertensive patients, south Ethiopia, 2022(n = 331).

Variables	Category	Frequency	Percent (%)
Marital status	Married	264	80.0
	Unmarried	18	5.4
	Divorced	19	5.6
	Widowed	30	9.0
Sex	Male	180	54.3
	Female	151	45.7
Religion	Orthodox	120	36.2
	Muslim	146	44.1
	Protestant	34	10.2
	Catholic	31	9.5
Age	18–39	128	38.6
	40–59	162	49.0
	≥ 60	41	12.4
Educational status	Illiterate	179	54.0
	Primary school	73	22.0
	Secondary school	37	11.0
	High school above	42	13.0
Occupation	Farming	102	31.0
	House wife	90	27.0
	Private business	92	28.0
	Government	39	12.0
	Daily labor	8	2.0
Wealth index			10.5
	Poorest	142	43.0
	Poor	85	32.0
	Medium	64	25.0
	Wealthy	40	12.0
Residence	Urban	139	42.0
	Rural	192	58.0
Family size	1–3	61	18.4
	4–6	172	52.0
	Above 6	98	29.6
Received nutritional education	Yes	140	42.0
	No	191	58.0
Have your family/friends help you to follow your recommended diet	Yes	89	27.0
	No	242	73.0

About 58.0% of the participants did not receive information on healthy eating, and the majority of the participants, 73.0%, had no family member or friends who supports them to adhere to their recommended dietary habits (Table 1).

### Dietary habits and patterns of respondents

Most of the respondents (57.0%) had consumed cereal-based foods more than three times per week while 193(58.5%) and 165(50.0%) consumed roots and tubers, and legumes more than three times per week, respectively. The majority of the respondents, 232(70.0%) had reported to consume vegetables, and fruits for more than three times per week. Furthermore, more than half of them, 206(61.0%) reported to consume milk and milk products for more than three times a week while only 158(48.0%) of respondents reported to consume any animal-source foods for at least three times per week. Out of the respondents, only 20(6.0%) do not drink coffee whereas 311(96.0%) drink coffee for at least once in a week. The majority 228(68.0%) reported that they do not have habit of drinking alcohol while 104(32.0%) drink at least one times per week (Table 2).

**Table 2:** Major dietary consumption patterns of hypertensive patients south Ethiopia Wolkite 2022(*n* = 331).

Food groups consumed	Frequency of consumption per week; frequency(percent)			
	Never	1–2 × per week	3–4 × per week	5–7 × per week
Cereals and grains	115(35.0)	27(9.0)	69(21)	120(36.0)
Roots and Tuber	113(34.0)	25(7.5)	45(13.5)	148(45.0)
Legumes	126(38.0)	40(12.0)	50(15.0)	115(35.0)
Fruits	63(19.0)	38(11.0)	45(14.0)	185(56.0)
Vegetable	68(21.0)	31(9.0)	63(19.0)	169(51.0)
Milk and milk products	93(29.5)	32(9.5)	79(23.0)	127(38.0)
Meat, fish and poultry	128(39.0)	45(14.0)	95(29.0)	63(19.0)
Fats and oil/ Plant based/ oil/liquid/ for cooking	217(66.0)	12(4.0)	17(5.0)	85(25.0)
Sweet and sugar /soft drinks/ sweetened beverages	105(32.0)	57(17.0)	36(11.0)	133(40.0)
Hot drinks /coffee/	20(6.0)	7(2.0)	7(2.0)	297(90.0)
Alcohol	227(68.0)	25(8.0)	33(10.0)	46(14.0)

To characterize the dietary consumption of clients in a better way, we employed a sequentially applied factor analysis. First, we have grouped foods into key food groups for factor analysis. Hence, we have checked for the presence of significant correlation, sample adequacy, and communality, and those food items not fulfilling these criteria were excluded step by step. In this study, we found a significant correlation with Bartlett's Test of Sphericity (X2 = 91; *p*-value < 0.0001) and a KMO value of 0.624. We computed the factor scores using the Bartlett method and ranked them into terciles as "low," "medium,” and “high” terciles of consumption. We derived five major dietary patterns, namely “cereals, fruits, and animal-source products," “legumes, vegetables, and milk products," “vegetable-source foods," “substances and alcohol drinks,” and “vegetable oils," explaining 62.3% of the total variance. The terciles of dietary consumption patterns are presented in Table 3.

**Table 3:** Centiles of major dietary pattens of adult hypertensive patients on chronic follow up in Southwest Ethiopia.

Dietary Pattens	Percentiles			
	Low	Medium	High	Variance explained by a factor
Dietary Pattern I: cereals, fruits, and animal-source products	109(33.2%)	110(33.5%)	109(33.2%)	14.0%
Dietary Pattern II: legumes, vegetables, and milk products	110(33.2%)	110(33.5%)	109(33.2%)	12.3%
Dietary Pattern III: vegetable-source foods	109(33.2%)	110(33.5%)	109(33.2%)	12.1%
Dietary Pattern IV: substances and alcohol drinks	111(33.2%)	109(33.5%)	108(33.2%)	12.5%
Dietary pattern V: vegetable oils	110(33.2%)	110(33.5%)	109(33.2%)	10.5
Bartlett's Test of Sphericity	X2 = 91; *p*-value < 0.0001			
Kaiser Mayer Olkin (KMO)	0.624			
Total variance explained (%)	62.3%			

### Prevalence of overnutrition

In this study, the overall prevalence of overweight and obesity among the study population was 95(29.0%), of which 72(22.0%) were overweight and 23(7.0%) were obese. On the other hand, Aa total of 38 (11.3%); 95% CI 8.1–15.2) of hypertension patients were found to be undernourished. With regard to estimated population prevalence, the prevalence of overnutrition, overweight, and obesity would be 24.1–34.2%, 17.6–26.6% 4.5–10.3% at 95% confidence level. Similarly, the prevalence of undernutrition would range from 8.1 to 15.2% among hypertension patients in the study area (Table 4).

**Table 4:** Nutritional status of hypertensive patients in southwest Ethiopia.

	Categories	Frequency	Percent (%)
Nutritional status based on BMI in kg m^−2^	Underweight	38	11.3
	Normal	196	59.8
	Overweight	72	22.0
	Obese	23	7.0
Overnutrition status	Underweight	38	11.3
	Normal	196	59.8
	Overnourished	95	29.0%
Mean BMI (Sd)	23.0 ± 4.3 kg m^-2^		

### Factors associated with overnutrition; OLR model

After considering all potential predictors, we assessed the proportional odds assumptions or the test of parallel lines. In summary, the chi-square distribution indicated that the proportional odds assumption was satisfied, and there wasn't enough evidence to reject the null hypothesis, suggesting that the proportional assumption holds (Table 5).

**Table 5:** Outputs for test of parallel line: Proportional odds assumption for ordinal logistic regression after incorporating relevant predictors in the final model.

	Test of parallel line: Proportional odds assumption			
Model	−2 Log Likelihood	Chi-Square	df	Sig
Null Hypothesis	337.740			
General	321.053	16.687	17	0.476

We have designed the multivariable ordinal logistic regression model in a backward, step-wise manner. The final model showed significant improvement compared to the intercept-only model (*p*-value = 0.01). Model fitness was further checked using the Deviance (X^2^ = 524; df = 507 *p*-value = 0.288) and Pearson (X^2^ = 476; df = 507 *p*-value = 0.829), indicating a fit model. Moreover, the predicted odds of overnutrition were found to be higher among males (AOR = 2.85; 95% CI 1.35–6.02), married (AOR = 1.47; 95% CI 0.69–3.12), and illiterates (AOR = 2.09; 95% CI 1.18–3.72), where the risk was higher by 2.85, 1.47, and 2.09 times as compared to their counterparts, respectively (Table 6).

**Table 6:** Multivariable ordinal logistic regression output showing factors associated with over nutrition among adult hypertension southern Ethiopia Wolkite 2022(n = 331).

Variables	Categories	Nutritional status (ordered)			AOR with 95% CI	*p*-value
		Undernourished	Normal	Over nourished		
Age in years	18–40	13	19	11	1	
	41–60	19	121	54	1.67(0.61–4.61)	0.321
	> 60	5	56	30	1.12(0.37–3.41)	0.843
Sex	Male	16	111	59	2.85(1.35–6.02)	0.006
	Female	21	85	36	1	
Marital status	Married	26	165	78	1.47(0.69–3.12)	0.136
	Unmarried	11	31	17	1	
Educational status	Illiterate	18	109	52	2.09(1.18–3.72)	0.012
	Literate	19	87	43	1	
Occupation	Government	3	15	11	6.83(1.19–39.2)	0.031
	Private	23	107	60	1.92(0.86–4.28)	0.109
	Farmer	11	74	24	1	
Residence	Urban	13	71	52	4.06(1.76–9.36)	0.0001
	Rural	24	125	43	1	
Family support	Yes	6	44	29	2.04(0.85–4.90)	0.109
	No	31	152	66	1	
DP-1; cereals, fruits, and animal-source products terciles	Low	7	71	31	1.56(0.72–3.34)	0.258
	Medium	19	62	29	0.86(0.41–1.81)	0.698
	High	11	63	35	1	
DP-3; vegetable-source foods terciles	Low	14	59	36	1.35(0.65–2.79)	0.422
	Medium	8	72	29	1.47(0.72–2.96)	0.288
	High	15	65	30	1	
DP-4; Substances and alcohol drinks terciles	Low	11	60	38	1.46(0.71–3.02)	0.308
	Medium	13	64	33	0.88(0.44–1.75)	0.706
	High	13	72	24	1	

Hence, we found that sex, residence, educational status, occupation, and residence of hypertension patients were significant predictors of overnutrition. Moreover, dietary consumption patterns were also very predictive of the odds of overnutrition, though they were not statistically significant. The predicted odds of being in overnutrition were higher among those 41–60 years old (AOR = 1.65; 95% CI 0.61–4.61) and above sixty years old (AOR = 1.12; 95% CI 0.37–3.41), indicating that the risk of overnutrition rises along with age. But still, the huge risk concentrates among middle-aged older adults (41–60 years; 67%). More importantly, the predicted odds of overnutrition were significantly higher for overnutrition employed (AOR = 6.83; 95% CI 1.19–39.2) and private workers (AOR = 1.92; 95% CI 0.86–4.28) as compared to farmer counter parts. Related to these, the predicted odds of overnutrition were found to be higher among urban dwellers (AOR = 4.06; 95% CI 1.76–9.36) (Table 6).

Concerning the association between dietary consumption patterns and the odds of overnutrition among hypertension patients, the odds of overnutrition were shown to be higher for those with lower consumption of cereals and animal-source foods, vegetables, and alcohol. For dietary patterns one and four, the odds of overnutrition were low for those with optimal (medium) consumption of these foods. Hence, the predicted odds of overnutrition were higher among those in the lower (AOR = 1.35; 95% CI 0.65–2.79) and medium (AOR = 1.47; 95% CI 0.72–2.96) terciles of vegetable-rich foods as compared to those in the higher terciles of consumption. The odds of overnutrition were also found to be higher among those with a lower tercile (AOR = 1.56; 0.72–3.34) of cereals and animal-source foods. Similarly, the odds of overnutrition were higher among those who had low and higher consumption of cereals and animal-source foods, implicating optimal intake in the median category and a lower risk. However, the risk was low among those with medium consumption of these food groups. This was similar for dietary pattern 4, where the risk was low among patients in the medium consumption classes (Table 6).

## Discussion

This study was to explore the magnitude and factors associated with overnutrition among hypertensive patients, where such evidence is lacking in Ethiopia. This study specifically contributed a lot to understanding the role of dietary consumption in a robust manner and their role in the odds of overnutrition, the known risk of adverse complications, and the occurrence of morbidities. Overall, we found that 29.0% of hypertension patients on follow-up had overnutrition, while 7.0% of the hypertension patients were obese. A review done for the adult general population in Ethiopia showed a pooled prevalence of overweight and obesity of 20.4% and 5.4%, respectively^25^. On the contrary, the overall prevalence of overnutrition is lower than the prevalence of overnutrition reported from Addis Ababa^26^ Gondar^27^ and Durame^28^ (44.6 and 9.6%). More specific studies showed a considerably higher prevalence of 36.3% (Nigeria) to 82.1% (Kenya), where a higher burden of obesity was reported, respectively. These are mainly due to urbanization and associated lifestyle changes, according to later studies^26^^,^^27^. These could be partly attributable to good adherence to the treatment plan and better fruit, vegetable, and cereal consumption. One review study conducted in the context of sub Saharan Africa showed that 57% of hypertension patients had abdominal obesity^20^.

In the present study, overnutrition risk was higher among males and in urban settings. Although direct evidence among hypertensive patients is lacking, evidence from the general adult population showed that females are at higher risk of central obesity (AOR = 5.59, 95% CI 2.95–10.57)^29^. This could be due to physiological factors, lack of exercise, environmental factors, and genetics^30^. In addition, there may be an obvious difference in dietary habits, physical activity, and lifestyles where the risk of overnutrition is higher among male and the contribution of central adiposity could be higher among males as well^31^. These could indicate only where the potential cases are concentrated, even though these characteristics are nonmodifiable. Furthermore, the increased risk of urbanization is mainly linked to unhealthy lifestyles, a tendency to consume processed, high-calorie foods, and limited activity-related expenditures. Evidence showed that men residing in urbanized cities in Ethiopia ((AOR = 1.8; 1.1–2.9) had 80% odds of increased risk for overnutrition^32^. The finding is also confirmed by previous studies. This could be related to the fact that urban residents tend to have an unhealthy diet with limited physical activities that increases the risk of overnutrition^33^ as compared to those from rural areas involved in physically demanding work such as agricultural activities^34^.

Literacy could potentially affect the odds of overnutrition. In our study, illiterate patients were found to be at risk, while previous studies showed the opposite. The link between literacy and overnutrition could be a double sword, where illiteracy and higher literacy could affect each other in various ways^32,35^. A study based on the national data also showed that the risk increase along with educational level (AOR = 3.6, 95% CI 2.1–6.2)^32^. Hence, illiteracy could greatly limit access to healthy foods and affect the selection of modern yet unhealthy diets. These might also be associated with early malnutrition and the later risk of obesity and metabolic syndrome. It has been observed that the patterns of overnutrition and non-communicable disease change over time, with illiterates and low socioeconomic groups becoming victims of the worst outcomes^36^.

The risks of overnutrition were higher for those with infrequent vegetable consumption patterns. This finding is supported by previous studies in Ethiopia^37,38^. Thus, eating non-starchy vegetables and fruits like apples, pears, and green leafy vegetables can promote weight loss and maintain optimal weight status^39^. This is due to the fact that vegetables are low in calories and allow a person to maintain satiety and energy balance^40^. The increased odds of overnutrition were associated with either low or high consumption of foods rich in cereal and animal-source foods. This finding is supported by a previous study^41^. These food groups are major sources of energy in the form of carbohydrates, proteins, and fats where excess consumption could lead to adiposity and poor blood pressure control^42^. While the increased risk among the lower terciles might be due to intentional dietary restriction due to pre-diagnosed overnutrition and risks. However, it should be noted that cereals are fiber-rich foods, which may encourage good gastrointestinal health and decrease the risks of excess cholesterol and obesity^43^. It is important to note that optimal dietary exposures are crucial for optimal health. On the contrary, low dietary exposure to nutrient-dense foods could also determine the risk of the late onset of overnutrition and chronic diseases^37^.

The current study brings novel evidence to one of the unresearched areas, although no studies have been conducted among hypertension patients. The use of dietary pattern analysis would make the current study predictive and informative^44^, where the majority of previous works rely on food group intake or dietary intake estimates. Moreover, the use of an advanced and more appropriate statistical approach would make it more informative, though with inherent limitations. For instance, the validity of BMI in indicating body fatness could be limited in indicating the actual risk of central obesity, where the use of waist circumference might be very predictive^45^. The items in the FFQ are limited in number and might not be exhaustive enough to capture them all. It is also very difficult to create a temporal relationship between diet and overnutrition; the diet might change after starting treatment while the outcome has already developed. Lastly, ordinal data assumes that the categories have a meaningful order and that the intervals between categories are equal. However, in the case of BMI categories, the intervals between categories may not be consistent in terms of health risks or other factors. Therefore, treating BMI categories as ordinal may oversimplify the complexity of the relationship between BMI and health outcomes and may not accurately capture the nuances of this relationship.

## Conclusions

Overnutrition among hypertension clients is a major public health problem. The risks of overnutrition were found to be higher among males, urban residents, illiterates, government workers, and private workers. With regard to dietary exposures, lower consumption of vegetables and both excess and low consumption of nutrient-dense dietary patterns (cereals, fruits, and animal-source foods) were associated with increased odds of overnutrition that could be used to draft tailored dietary counseling and interventions. This increased occurrence of overnutrition could further threaten morbidity and mortality from complications of hypertension. This can be addressed via enhanced behavioral change models, dietary counseling, and other lifestyle interventions promoting healthy dietary consumption. Hence, contextualized dietary counseling is mandatory with the aim of achieving optimal intakes.

## Methods and materials

### Study area and design

A facility-based survey was employed among adult hypertensive patients at Wolkite University Specialized Hospital (WUSH) from July 05 to September 24/2022. The hospital is both a referral and teaching hospital, with 123 beds serving approximately 150,000 admissions and 80, 000 outpatient visits a year, targeting an estimated five million people in the catchment area. The hospital provides comprehensive care for about 1694 ambulatory hypertensive patients in addition to impatient care. The majority of the catchment population resides in rural areas, with agriculture being common. Moreover, consumption of "*enset*," fruits, vegetables, "*teff*,” and other staple crops is common in the study area. *Enset* is a plant commonly grown and consumed plant like banana plant while *teff* is staple crop to be consumed in the form of flat bread, bread, or other forms.

### Study population

The current study targeted all adult hypertensive patients (aged above 18 years of age) on chronic care at the hospital over the last year. Among these, randomly selected hypertensive patients who were currently on antihypertensive medication in the hospital from May to November 2022 were included. Those adults who were pregnant women, developed ascites (fluid accumulation in the abdominal cavity), and were critically ill were excluded, as the BMI measure tends to be biased.

### Sample size determination and sample selection

To estimate the minimum sample required for this study, we employed sample estimation method for single proportion and two proportion to estimate the overall prevalence of overnutrition and make comparison among groups. Hence, we considered a 95% confidence level, marginal error of 5%, 50% as estimated magnitude of overnutrition to maximize efficiency, and a 10% for non-response rate; the required sample size was 422. Then after finite corrections (*n* = 1694), a total of 338 sample of hypertension clients were studied.

To estimate the minimum sample required for this study, we employed the sample estimation method for single proportions and two proportions to estimate the overall prevalence of overnutrition and make comparisons among groups. Hence, we considered a 95% confidence level, a marginal error of 5%, 50% as the estimated magnitude of overnutrition to maximize efficiency, and a 10% non-response rate; the required sample size was 422. Then, after finite corrections (*n* = 1694), out of a total of 338, 328 hypertension clients were studied.

### Data collection methods

Data were collected by using face-to-face interviews using a structured questionnaire including basic demographics, treatment-related characteristics, and dietary consumption through a validated Food Frequency Questionnaire (FFQ) which was validated in Ethiopia^46^ and these tool was previously applied^37,38^.The FFQ was elicited over the past month to capture the usual dietary consumption after contextualizing it as per the national food composition table. The food items were taken from the Ethiopian Food-Based Dietary Guide (EFBDG)^47^. For each food item, participants were indicated their average frequency of consumption over the past month by checking 1 out of the 5 frequency categories. Each item was arranged (ranging from never, 1–2 times per week, 3–4 times per week, and 5–7 times per week. The tool was developed in English language and administered in local languages.

We employed a standardized anthropometric measurement of weight and height in accordance with the World Health Organization standard approaches for anthropometric measurements^48^. Weight measurement was done at the follow-up clinic using a calibrated electronic weight scale under light clothing without shoes to the nearest 0.1 kg (kg). The height measurements were done using a standing stadiometer (SECA Germany) and recorded to the nearest 0.1 cm. The height measurement was done while the client was in Frankfurt horizontal plane. These measurements were done twice, and the average of the two was recorded.

The data collection was done by trained health officers and supervised by nutritionists with basic skills in dietary surveys. A pre-test was done on 5% of the sample in another nearby hospital, and we made some changes to the tool and approach to collecting the data. Weight and height scales were calibrated. The measurements were done on a flat floor. We employed the performance of each anthropometric measurer during the pretest, evaluated for inter- and intra-observer variations, and compared against the standard. Those with unacceptable values were excluded from collecting data for our survey. One-day training was given for data collectors, and questionnaires were checked for completeness on a daily basis. Missed values and implausible responses and values were cleaned up.

### Variables of the study

The dependent variable of this study was overnutrition, measured by BMI. Overnutrition is defined when the BMI is above 25 kg/m^2^, with a value between 25 and 29.9 kg/m^2^ as overweight and above 30 kg/m^2^ as obese^49^. In addition, we assessed socio-demographic characteristics (sex, age, educational status, marital status, income, family size, occupational status, residence, number of people in the household, and monthly income), socioeconomic characteristics, dietary habits, dietary food consumption, substance use, and behavioral factors.

### Data processing and data analysis

The data was coded and entered into Epi Data Version 3.1. The data analysis was done in SPSS version 26.0 and STATA version 14. The normality of continuous variables was checked using a Kolmogorov–Smirnov test along with a *p*-value. We reported the mean and standard deviation for normally distributed variables. The results were described in frequencies and percentages and presented in statistical tables and graphs. Mean, standard deviation, and percentage describe the study population in relation to relevant variables. We have calculated the BMI using weight in kg divided by height in meters squared.

The wealth index was developed using principal component analysis of the dummy-coded asset variables adopted from the Demographic and Health Survey modules. We have collected data on the assets of a range of durable assets, such as cars, refrigerators, televisions, radios, materials for dwelling floors and roofs, toilet facilities, electricity supply, sources of drinking water, agricultural land and farm animals, and households owning a mobile phone^50^.

As the dietary pattern analysis allows us to capture the usual consumption in a more reliable way and is very predictive of disease outcome, we employed an exploratory factor analysis using principal component analysis to derive major dietary patterns. For this purpose, we have reorganized the FFQ food items and regrouped them into reasonable categories as per the EFBDG^47^. Assumptions for the appropriateness of factor analysis were checked using the presence of substantial correlations (Pearson correlation above 0.3 and a significant Bartlette Test of Sphericity), Kaiser-Mayer-Olkin for sample adequacy for the set of variables (> 0.5), and Bartlett’s test of sphericity (0.05). All assumptions were checked sequentially to come up with factor scores that were ranked and presented in the form of wealth quintiles for wealth index and dietary patterns (ranked as low, medium, and high for ease of presentation).

We employed a step-wise ordinal logistic model after setting up the variables for more clear presentations^24^. First, Brant test was conducted to assess the proportional odds assumption for ordinal logistic regression model. And a p-value above 0.5 is considered as fulfilled assumptions of ordinal logistic regression and this was done after including all potential predictors of overnutrition in the final model. We have included the relevant background variables and the major dietary consumption patterns derived from statistical procedures. Variables with a significant association with overnutrition, important variables from previous evidence, and biologically relevant risk factors were included in the final model. Moreover, the model fitness was checked via the log-likelihood method (*p*-value below 0.05), Pearson (*p* = 0.288), and Deviance goodness of model fitness (*P* = 0.829), indicting a fit model under the null hypothesis. The beta coefficients obtained from the final model were exported to MS Excel for the calculation of the adjusted odds ratio. We have checked multicollinearity using the standard error value above two and an unstable model with the addition or removal of additional predictors. Hence, we have reported the adjusted odds ratios along with 95% confidence intervals and the corresponding p-values. All statistical tests were considered significant at a p-value less than 0.05.

### Ethical approval and consent to participate

Ethical approval was sought from the Wolkite University Institutional Review Board (RCSUIL_C/056/14). We obtained written informed consent from each participant after explaining the study procedures. All the study methods and procedures were implemented in accordance with the approved procedures. The study was conducted in accordance with the Helsinki Declaration.

## Data Availability

All data generated or analyzed during this study are included in the submitted manuscript.

## Abbreviations

BMI: Body mass index
AOR: Adjusted odds ratio
BP: Blood pressure
CI: Confidence interval
EFBDG: Ethiopian food based dietary guideline
FFQ: Food frequency questionnaire
LMICs: Low- and Middle-income countries
OLR: Ordinal logistic regression
SD: Standard deviation
T2DM: Type II diabetes mellitus
WUSH: Wolkite university specialized hospital
